# A New Approach to Micromachining: High-Precision and Innovative Additive Manufacturing Solutions Based on Photopolymerization Technology

**DOI:** 10.3390/ma13132951

**Published:** 2020-07-01

**Authors:** Paweł Fiedor, Joanna Ortyl

**Affiliations:** 1 Faculty of Chemical Engineering and Technology, Cracow University of Technology, Warszawska 24, 31-155 Cracow, Poland; pawel.fiedor@doktorant.pk.edu.pl; 2 Photo HiTech Ltd., Bobrzyńskiego 14, 30-348 Cracow, Poland

**Keywords:** 3D printing, high-resolution, additive manufacturing, photopolymerization, industry 4.0, stereolithography, two-photon polymerization

## Abstract

The following article introduces technologies that build three dimensional (3D) objects by adding layer-upon-layer of material, also called additive manufacturing technologies. Furthermore, most important features supporting the conscious choice of 3D printing methods for applications in micro and nanomanufacturing are covered. The micromanufacturing method covers photopolymerization-based methods such as stereolithography (SLA), digital light processing (DLP), the liquid crystal display–DLP coupled method, two-photon polymerization (TPP), and inkjet-based methods. Functional photocurable materials, with magnetic, conductive, or specific optical applications in the 3D printing processes are also reviewed.

## 1. Introduction

Three dimensional (3D) printing is currently an extremely important branch of Research and Development (R&D) departments. This is because of its rapid prototyping, swift elimination of design errors, and improvements of the product at the prototyping stage. This approach significantly accelerates the implementation of new solutions without incurring significant production costs and eliminating in-production testing of underdeveloped models. Thanks to 3D printing techniques, making a prototype with complex geometry has become possible in a short time with unprecedented precision [1].

**Figure 1:**
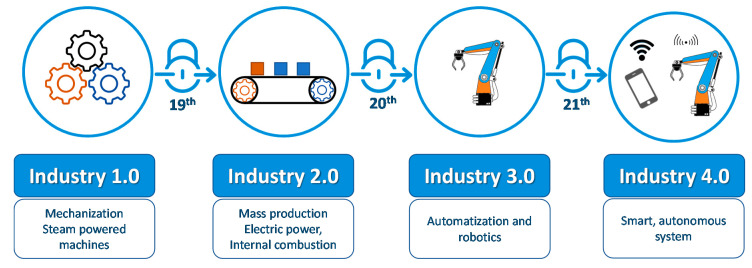
Industrial revolution—from Industry 1.0 to Industry 4.0. The concept of Industry 4.0 is based on encompassing a combination of traditional manufacturing and industrial platforms and practices with the latest smart technologies.

Progressive computerization of manufacturing processes introduces us to a new era called Industry 4.0. This level of smart production was enabled by significant breakthroughs in artificial intelligence, robotics, nanotechnology, and 3D printing observed in the twenty-first century. Thanks to the extreme customization and personalization of production technologies, the practice of Industry 4.0 is an observed phenomenon in every section of manufacturing processes. The application of artificial intelligence (AI) algorithms for the preparation and transformation of 3D models significantly quickens and improves the quality of 3D figures. AI has been successfully applied in printability checking, slicing acceleration, nozzle path planning, and, among others, cloud service platforms [2]. Evolution of industry models are presented in Figure 1.

The industry has just begun to adopt additive manufacturing methods, which they use mainly to prototype and produce individual items. With Industry 4.0, 3D printing methods will be widely used to produce small batches of made-to-order products that offer construction advantages, such as complex, lightweight designs [3].

The flexibility of 3D printing opens the way for sharing production capacities between diverse companies for better utilization of assets and supporting growth and will also open up opportunities to share production capacities between different companies, thereby better utilizing these assets [4]. Research projects such as Horizon 2020 are focused on creating such business models in industry.

Manufacturers have implemented 3D printing technologies through seven different additive manufacturing processes: powder bed fusion, vat photopolymerization, binder jetting, material extrusion, directed energy deposition, material jetting, and sheet lamination. Each method is achieved through a different variation of 3D printing technology, which varies based on its material state, sources of light or heat, print axes, feed systems, and post-production processing [5].

- Powder bed fusion: It works by melting powder to fuse particles together. It is ideal for most types of manufacturing [6].
- Vat photopolymerization: It uses a liquid instead of a powder or filament in its build platform and is a light-activated process. It is ideal for low-run injection molds [7,8].
- Directed energy deposition: It uses highly focused thermal energy delivered via a laser, electron beam, or plasma art to melt and fuse material. It is used exclusively in metal additive manufacturing [9,10].
- Material jetting: Tiny nozzles dispense droplets of a waxy photopolymer, layer by layer, which is hardened via UV light. It is ideal for items requiring high detail and high accuracy [11,12].
- Binder jetting: It is similar to material jetting but instead uses a powdered material and a binding agent. It is primarily used in furniture design models [13,14].
- Material extrusion: A thermoplastic filament is extruded through a heated nozzle onto the build platform, which solidifies as it cools. It is the most commonly referred to additive manufacturing method when someone is discussing 3D printing [15,16].
- Sheet lamination: Ultra-thin layers of solid material are bonded by alternating layers of adhesive. It is best used for non-functional models [17].

The history of 3D printing in its extremely turbulent beginnings revealed many ideas and solutions that have been verified by the market over time [18]. Among the ideas that have been developed for commercialized technologies based on the polymerization of liquid resin are thermal processing of thermoplastic materials, melting/sintering of metals, and methods creating prints from composite materials or rubber. The list of methods that should be highlighted in the topic of 3D printing is presented below [19]:

- Photopolymerization of liquid resin:
  o SLA—stereolithography,
  o DLP—digital light processing,
  o MJP—multijet printing,
  o BJ—binder jetting,
  o CAL—computed axial lithography,
  o SGC—solid ground curing,
  o CLIP—continuous liquid interface production.
- Thermal processing of thermoplastic polymers:
  o FDM—fused deposition modeling,
  o BPM—ballistic particle manufacturing,
  o SLS—selective laser sintering.
- Metal structure fabrication:
  o DED—directed energy deposition,
  o DMLS/SLM—direct metal laser sintering,
  o EBM—electron-beam additive manufacturing,
  o MBJ—metal binder jetting.
- Other methods:
  o LAM—liquid additive manufacturing deposits a liquid or highly viscous material (e.g., liquid silicone rubber) onto a build surface to create an object, which is then vulcanized using heat to harden it,
  o LOM—laminated object manufacturing patented by Michael Feygin—printers that cut cross-sections out of special adhesive coated paper using a carbon dioxide laser and then laminate them together,
  o CFF—continuous filament fabrication.

## 2. Photopolymerization-Based 3D Printing Methods

**Figure 2:**
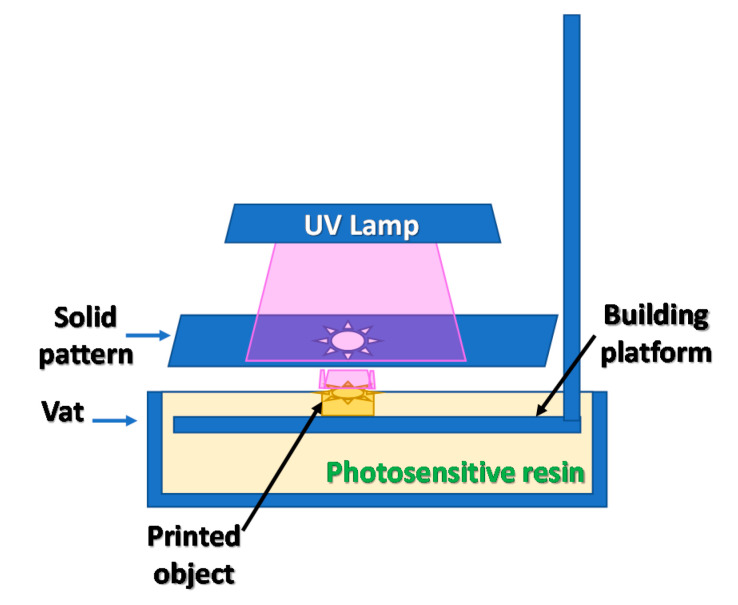
Origin of 3D printing; diagram of the principle of operation.

In the very beginning of 3D printing, photopolymers were applied for manufacturing designed structures [20]. To produce a three-dimensional element, flat templates with a shape corresponding to the element’s cross-section were used, through which the photosetting resin was illuminated [21]. The depth of the element was obtained in the same way as in today’s printers—by maneuvering the printer table up and down. The template-based solution found little widespread interest due to the limited possibilities of the formation of shapes, and the length of the process. A scheme of the machine used in this approach is presented in Figure 2. It became necessary to find a solution to obtain objects with a more complex geometry [22,23].

### 2.1. Stereolithography (SLA) Overview

**Figure 3:**
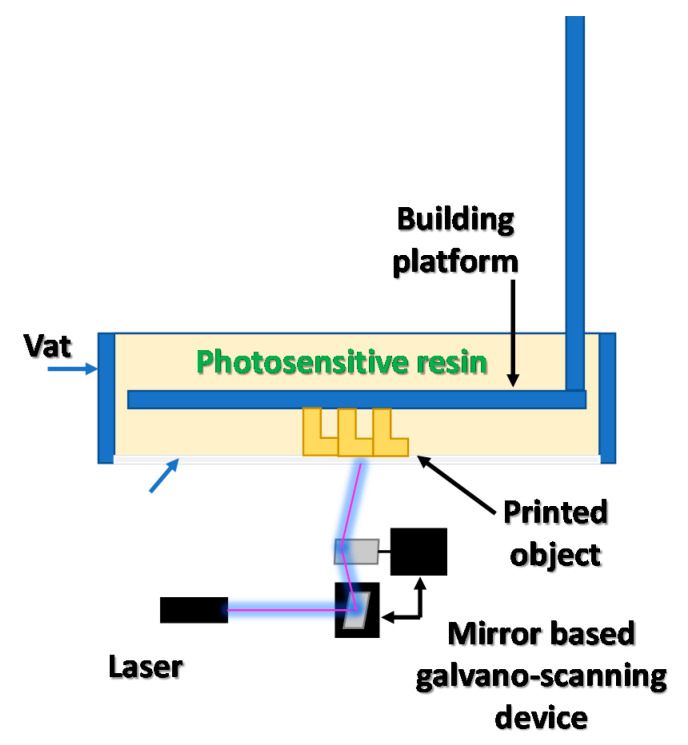
The modern design of the stereolithography system with a light-permeable membrane at the bottom of the resin vat.

The invention of the stereolithography (SLA) method by Chuck Hull is considered as the initial stage of 3D printing development. The SLA method is based on curing the liquid photosensitive resin with an ultraviolet laser beam. This approach allows the model’s geometry to change as its height increases, and as a consequence, to produce complex 3D elements in a single process. The SLA process of creating the printouts is based on scanning the photosensitive material with a UV laser beam—section by section, layer by layer—until a complete 3D print is obtained [24]. The basic scheme of the SLA 3D printer is presented in Figure 3. This approach required the creation of a data transfer method between the computer and the printing device (3D printer). Special STL (stereolithography) file formats were developed for this purpose [25,26].

### 2.2. Digital Light Processing (DLP) Overview

**Figure 4:**
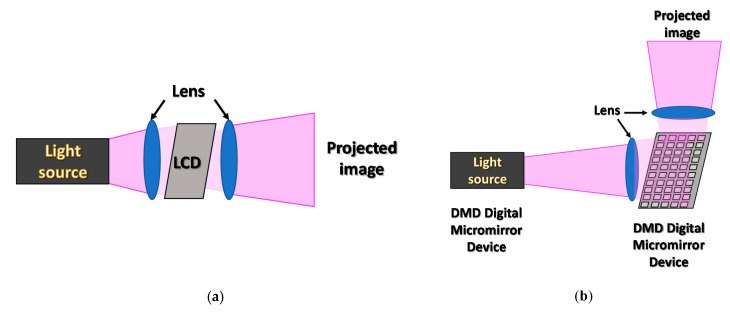
Principle of operation for (**a**) LCD and (**b**) DMD type projectors.

An important stage in the development of 3D printing techniques was the invention of the DLP (digital light processing) 3D printing process. In the DLP technique, the object’s cross-sectional shape is displayed by a digital projector. By displaying consecutive cross-sections of a printed model, subsequent layers of the printout with its changed geometry are created. Initially, the illumination of the photosetting resin was conducted from the top of the resin vat, while the resulting model was immersed in a liquid composition. This approach required significant amounts of photosetting material, and significantly limited the height of the print; hence, a method in which the bottom of the vat was made from transparent FEP (fluorinated ethylene propylene) adhesive film was developed [27,28]. Thanks to this arrangement, in DLP 3D printers, the amount of photosensitive material needed for an operation is minimized, and the height limit of printouts has been overcome, because the printout itself is “pulled out” from the liquid resin bath. In the case of the DLP process, the resolution of models depends on the design of the projector and the optics used to display the cross-sectional images. The main disadvantages of DLP are the resolution and light intensity, which decrease with the size of the projected scene as is observed in all projectors [29]. The lamps used in the projectors emit light with a wide spectrum, but only a small part of the energy radiated by the lamp falls into the range below 400 nm in which most photoinitiators are active. A schematic representation of an LCD-based projector is shown in Figure 4a [26,30].

The main type of projectors used in the early development of this technique were projectors equipped with a matrix of many microscopic mirrors (DMD digital micromirror device) [31]. The micromirrors’ motions were controlled by the projector software to map the exact shape of the object’s cross-section. The factor limiting the resolution of the system was, of course, the number and size of the mirrors and the size of the displayed image. The amount of light reaching the resin vat was limited because the high intensity of light illuminating the DMD matrix caused reflector burnouts and increases in the matrix temperature and resulted in artefacts in the displayed image. A DMD-based projector scheme is presented in Figure 4b [32].

### 2.3. LCD 3D Printing Overview

In the next approach, instead of a matrix of micro-mirrors, an LCD screen was used. An LCD matrix was placed on the optical path between the lamp and the vat with the photosensitive material. This system, with a proper beam forming lens, creates an object profile in the resin. Such a system is usually characterized by a higher resolution than DMD and is called the LCD–DLP system [33]. In the case of LCD matrices, the amount of energy supplied to the photosetting resin is limited by a high absorbance of the LCD matrix itself. The absorbance of the matrix material changes with the wavelength of supplied light and almost completely blocks radiation below 400 nm [34].

**Figure 5:**
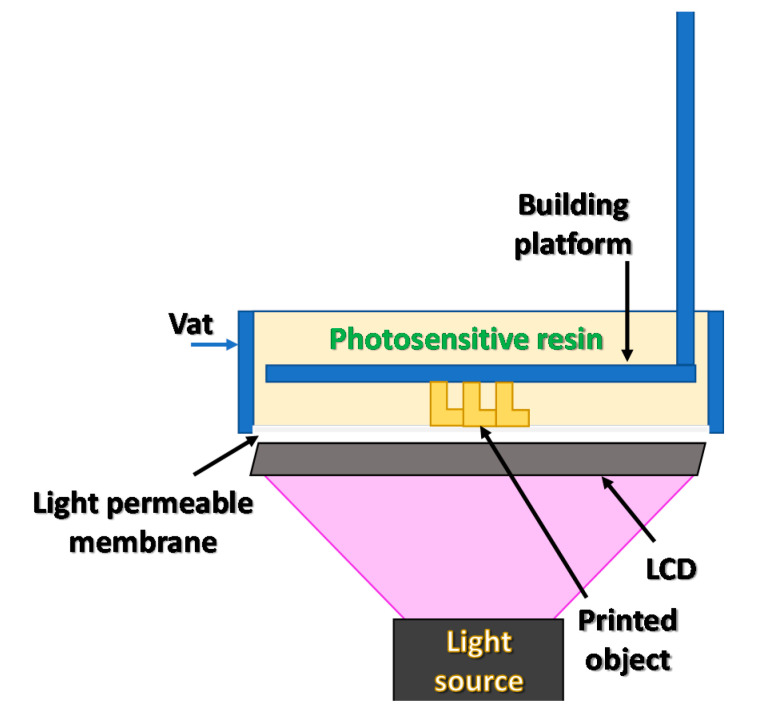
A schematic representation of an LCD–digital light processing (DLP) 3D printer.

For this reason, modern printer designs use LED lamps with a wavelength of 405 nm or higher. The amount of light is still small in relation to the intensity of the lighting used. This problem reduces the photopolymerization of materials that require high light intensity. A graphical scheme of an LCD–DLP 3D printer is shown in Figure 5 [21].

### 2.4. Continuous 3D Printing Overview

The next stage in the development of the 3D printing processes is continuous printing CLIP (continuous liquid interface production). In the CLIP process, the photosensitive material is illuminated by cross-sections of the model with a smooth transition between them, and movement of the printing table is smooth and resembles the slow draw of the model from a resin vat. The high reactivity of the photosetting resin, as well as the intensity of lighting, is of great importance in this approach [35]. A most important factor for continuous 3D printing is the material from which the bottom of the vat is made. This material must be light-permeable and must prevent polymerization of the material on the resin’s contact surface. For this purpose, oxygen permeable membranes are used. Their task is to prevent polymerization on their surface by oxygen inhibition of free radical polymerization. Oxygen inhibition is effective only a short distance from the membrane, while oxygen diffusion into the deeper layers of resin is hindered so that free radical photopolymerization can take place [36].

However, this method has its limitations; it is not suitable for printing elements containing large flat horizontal surfaces. Viscous resin combined with continuous movement creates a huge suction force between the bottom membrane and the printout when pulling the model out of the liquid resin. This suction force causes deformation of the oxygen permeable membrane and distorts the model [37].

### 2.5. Inkjet Printing

3D printing technology operating on a principle of shooting tiny droplets of material through microscopic nozzles towards the substrate has been popularized as a tool in traditional inkjet printers. In the late 1980s and beginning of 1990s, at the Massachusetts Institute of Technology (MIT), a 3D printing method using an adhesive agent applied by an inkjet head to layers of powder material was developed. In the same year, this method was patented and adopted the name binder jet printing, joining the methods of additive manufacturing [38]. The method for the formation of 3D elements consists of applying microdroplets of the binder by the inkjet head onto a layer of powder material substrate. After applying the binding agent, the printing area is covered by another layer of powder and the process is repeated layer by layer until a complete printout is obtained [39,40]. As a building material in the binder jetting method, materials of various characteristics can be used, from polymer powders through to ceramic and metallic materials, while solvent and photosetting adhesives can be used as binders [13,14,38,41]. After the printing process, the material can be immediately ready for use (polymer powders) or subjected to a high-temperature sintering process (ceramic and metallic powders) in order to burn out the binding agent and solidify the powder material. Despite the variety of materials and binders that can be used for printing in this method, its resolution is limited and reaches from 25 to 50 μm [42].

**Figure 6:**
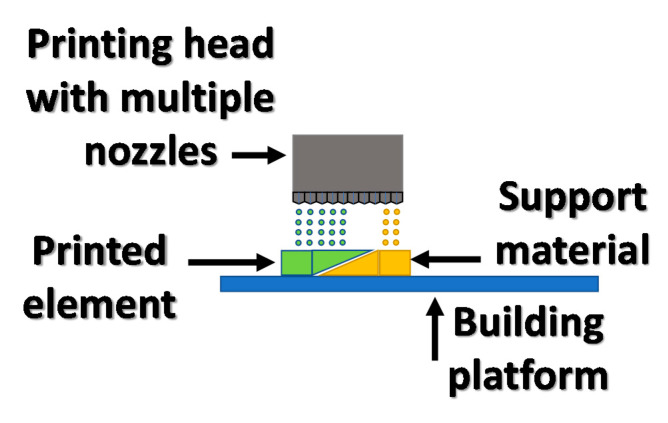
Inkjet 3D printing scheme of method operation.

A variant of ink-jetting 3D printing with the widest commercial application is the method using photosetting materials from which the exact element is created; however, elements with more complicated geometry require a second material that will support overhang layers of the printout. A method based on the inkjet system using photo-polymerization is called multijet printing (MJP). In Figure 6, the basic MJP system is presented. In the multijet printing technique, a wax material is used as a support in addition to a photosensitive resin. Multijet printing involves applying a layer of photo-curable material and support material to a flat platform and then crosslinking the material by a UV light. After creating an entire printout, the wax support material is removed by melting it at increased temperatures. An additional advantage of the inkjet method is the ability to simultaneously print with several different materials, which allows the creation of color prints with a reasonably high resolution. This method is currently widely used commercially in design and prototyping processes with a 50–100 μm accuracy [38].

The family of inkjet technologies also includes a 3D printing method based on applying layer by layer of ink containing nanoparticles of functional materials dispersed in a solvent or photopolymer. As the ink consists of nanoparticles suspended in a solvent, the printout is made by applying drops on the substrate and then evaporating the solvent. However, this approach suffers from a resolution limitation resulting from the size of the nozzle. The nozzle’s size must, however, remain larger than the diameter of the particles in ink to prevent it from clogging. This limits the resolution of elements to the micrometric level [43].

**Figure 7:**
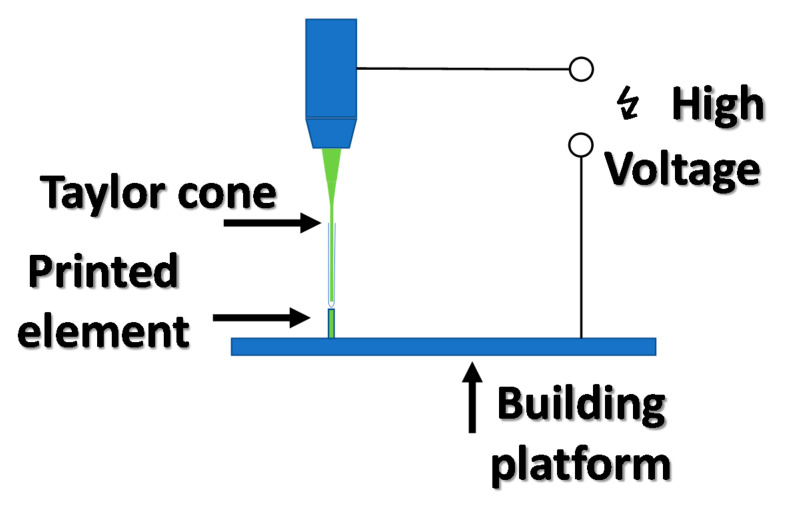
Electrohydrodynamic inkjet printing principle in operation.

Increasing the method resolution below the nozzle size is possible by applying a potential difference of several kV between the nozzle and the substrate. High electrical potential differences force the drop formed on the printer nozzle, which is drawn towards the ground, creating a Taylor cone. At a certain voltage applied between the nozzle and the ground, the formed Taylor cone reaches the platform, creating a droplet much smaller than the nozzle diameter. This method is called EHD MJP— electrohydrodynamic multijet printing—and allows the production of components with nanometric sizes. This method was used for inkjet printing, with 0.1% gold nanoparticle solutions, to create printed gold electrodes with a width of 80 to 500 nm [44]. The electrohydrodynamic MJP printing setup is shown in Figure 7.

A modification of the EHD MJP method, which allows the precise control of the voltage applied to the system, is the use of lithium niobate crystals as pyroelectric elements. The rapid increase in temperature of the lithium niobate crystal causes the charge to deposit on the crystal’s surface. Heating LiNbOx by 100 °C creates a potential difference of about 10 kV, which is the value necessary for printing by the EHD MJP method and allows for very precise control over the process [45,46].

### 2.6. Computer Axial Lithography

**Figure 8:**
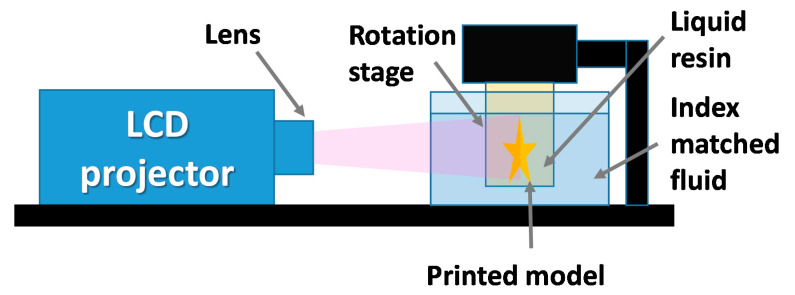
Computer axial lithography setup.

A novel and highly versatile method based on photopolymerization is computed axial lithography (CAL). Unlike others 3D printing methods, CAL is based on volumetric photopolymerization of the whole model, instead of building a model through deposition of material layers. This approach is possible due to different mechanism of model cross-section preparation than in DLP or SLA methods [47]. The CAL method utilizes a DLP projector to display horizontal images of the printed model at different angles onto the side panel of the vat. In the CAL method, two liquid media are present, an external liquid with refraction index matched with the second media, a proper photo setting resin inside a smaller rotational vat. The small resin tank is a printing platform that rotates with an angle coupled to the angle of the displayed image. Computed axial lithography offer great advantages in comparison to other photopolymerization-based methods, such as printing speed and the possibility of incorporating objects into 3D printed models. An operational scheme of the CAL process is shown in Figure 8 [25].

### 2.7. High Area Rapid Printing

The high area rapid printing (HARP) method is another method of additive manufacturing focused on enhancing printing speed and passible volumes of models. Vertical printing speeds in the HARP method reach 430 mm per hour with processing volumes up to 100 L. The light processing unit utilized for the photocuring process is based on a high power UV digital light processing unit. The main advantage of this method is application of fluorinated oil instead of fluorinated olefin membrane at the bottom of the resin vat. Thanks to the application of fluorinated oil, during the curing process adhesive forces between the oil layer and the emerging solid part are very small, which allows printing without detaching the model from the bottom membrane. The moving fluorinated oil help also with dissipation of heat released during photopolymerization of huge models. Heat transfer from the photocured model, according to the authors of the HARP method, was the main factor limiting size of the model and its creation speed [48].

### 2.8. Hot Lithography

A new approach for high speed 3D printing methods is hot lithography. Hot lithography, by increasing temperature of resin in the vat, reduces the viscosity of the media and opens the way for application of higher molecular weight resins [49]. Replacing traditional low viscous resins with more viscous photopolymers benefits in enhancements of final mechanical properties of resulting models. Higher temperatures of photocurable media results also in reduction of critical energy E_0_ (critical exposure) of resin, which result in increasing polymerization speed at the same irradiation power. By application of hot lithography, functional parts with over 60 MPa tensile strength and 20% elongation at break are printed, which is very difficult to achieve for low viscous photocurable resins. Furthermore, green parts (before post curing process) printed at elevated temperatures show higher conversion and better mechanical properties, which helps for cleaning and post-processing of printed parts [27]. Depending on the photopolymer used, the vat temperature can reach from room temperature up to 120 °C [50].

### 2.9. Liquid Bridge Microstereolithography

**Figure 9:**
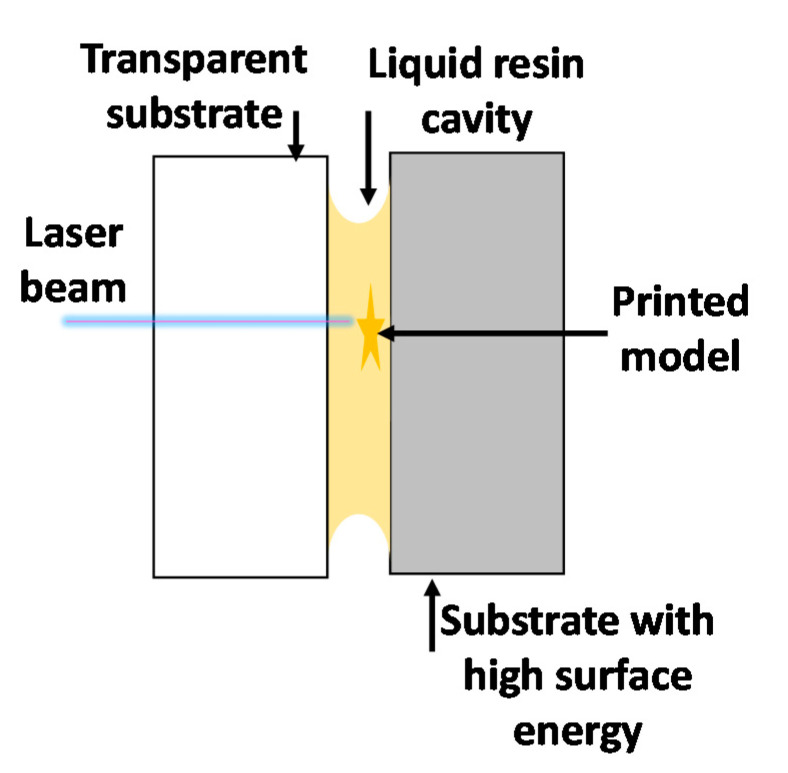
View of liquid bridge microstereolithography (LBMSL) setup.

Liquid bridge microstereolithography (LBMSL) [28] is a variant of the stereolithography method for manufacturing objects at the micro scale. The main advantage of this method is that it does not require a vat with a significant amount of photosetting resin, because the entire 3D printing process is carried out by a liquid bridge formed between two flat surfaces by the surface tension of the photosetting resin. One of the flat surfaces involved in LBMSL gives great adhesion to printed elements and holds printed structures together, while the second (transparent) substrate with low adhesion to resin can be easily removed after the printing process. The LBMSL method is advantageous due to small amounts of resin required for the printing process, constraining the material surface, which drastically reduces oxygen inhibition. The principle of liquid bridge microstereolithography is presented in Figure 9 [51].

### 2.10. Direct Ink Writing

Direct ink writing (DIW) is an additive manufacturing method that employ a computer-controlled translation stage, and an ink depositing nozzle, which applies photosetting material on the building platform. With this method, different material can be utilized as an ink, from tough and rigid resins, to rubber-like polymers and silicones, to different composite materials. Resolution of the DIW method is dependent mostly on the size of the nozzle, which can be as small as 30 μm. Direct ink writing was successfully employed for fabrication of microfluid devices such as branched channels, mixers, chambers, and droplets generators [29].

### 2.11. Large-Scale Metamask-Assisted 3D Fabrication

**Figure 10:**
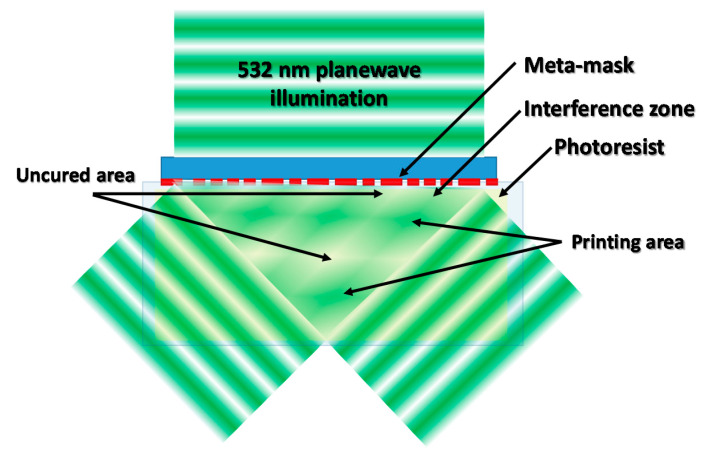
Creation of diffraction pattern by meta-mask setup involved in 3D printing process.

Metasurface masks can generate complex intensity distributions based on creation of interference patterns, for fast and high resolution 3D printing. Large-scale metamask-assisted 3D fabrication method use photoresists, such as SU-08 with a photoinitiating system active over 500 nm, as a building material. The light source used for fabrication of 3D structures is a 532 nm laser with linear polarization. This method of light intensity modulation can be utilized for formation of structures periodic in three dimensions. The metamask-assisted 3D printing is a fast, large-scale, and robust system for manufacturing 3D structures, but it main disadvantage is fabrication of periodic structures, which refer to diffraction patterns generated by metamask. A diagram of diffraction pattern formation, utilizing meta-mask for refraction of light, is presented in Figure 10 [15].

## 3. High Accuracy Photopolymerization-Based 3D Printing Processes

### 3.1. Preparation of Projects for the 3D Printing Process

Each of the 3D printing methods requires preparation of an appropriate set of instructions for the 3D printer. These instructions vary depending on the type of printer and printing technique used, but they have a common element—the 3D model. The 3D models are created using CAD (computer-aided design) software, but for processing them into a set of instructions understood by a 3D printer, the STL file format (abb. from stereolithography) was adopted as the standard.

In the STL format files, the geometry of the 3D object is saved as a mesh of polygons forming all its surfaces. For communication with the 3D printer, it is necessary to prepare a set of commands that the printer is to perform based on the geometry read from the STL file. To do this, programs called slicers were developed; their task is to divide the existing 3D model into a series of cross-sections according to the parameters set by the operator. Printouts in technologies using laser heads or printheads require an additional division of the model’s cross-sections into paths along which the printhead will follow. For methods where the printing process is based on following paths by the printhead, spatial resolution highly depends on parameters set up in slicer software, such as path width and height. Path width defines horizontal (x–y plane) resolution, while path height gives values of vertical (along *z* axis) resolution. Decreasing values of path height and width result in better shape accuracy of printing elements for price of increasing printing time. The output file, after the slicing operation, presents a set of commands, understood by the printer driver, saved in * gcode text file format [41].

**Figure 11:**
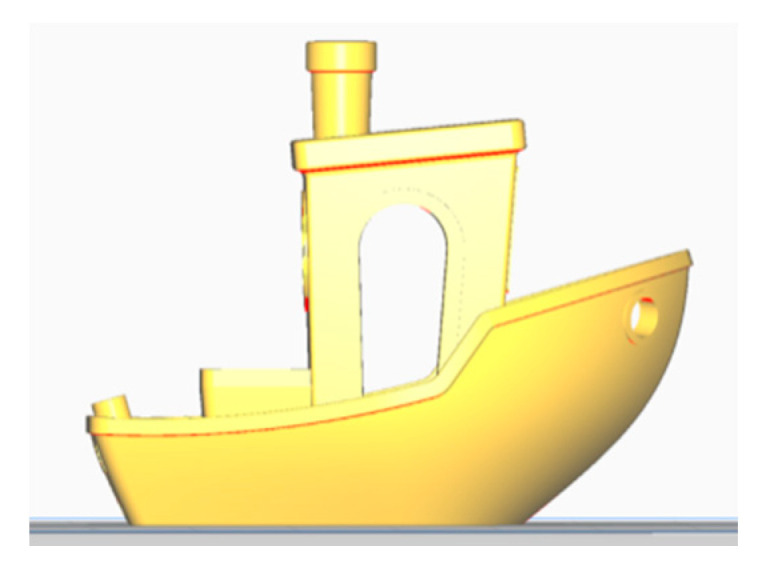
Model of a “Benchy” boat form *.stl file.

**Figure 12:**
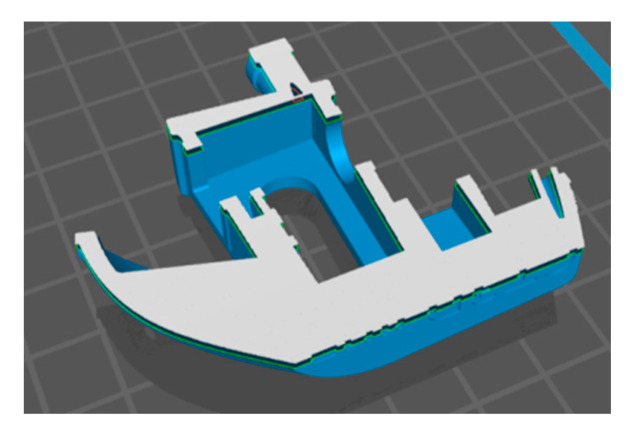
“Benchy” model sliced and prepared for the DLP method.

**Figure 13:**
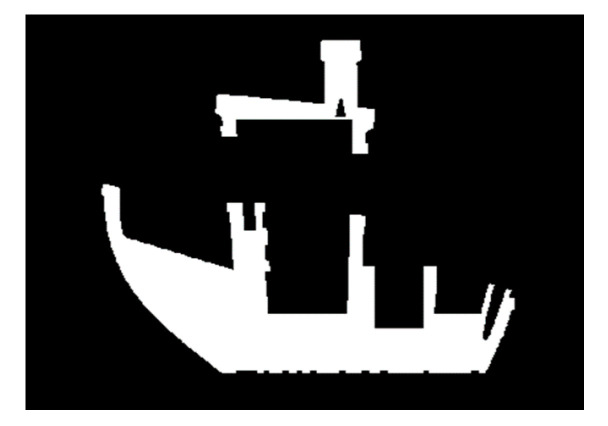
Single cross-section of a “Benchy” model—view displayed by an LCD screen in the LCD–DLP method in the middle of the print.

Having these sets of commands, the printer is able to reproduce a geometric object with the use of an appropriate material; however, depending on the design of the device and quality of the machine code, the obtained 3D printout result may have a different spatial resolution. An example model preparation process for DLP 3D printing is shown in Figure 11, Figure 12 and Figure 13.

### 3.2. Future Fabricated with Photopolymerization

Different 3D printing methods require the use of resins with different parameters. For resins photosensitive in the 365 nm–450 nm range, important parameters include viscosity, absorption properties of the initiator and the resin itself, and also critical resin exposure. The absorption properties result in an effective depth of light penetration, and critical exposure regulates the amount of energy that must be supplied to the resin to initiate the polymerization process [52].

The type of resin significantly affects the properties of the print. Today, commercial resins are available in variants that allow the model to be printed with properties ranging from elastic and flexible, through to brittle and soft wax materials used in foundries, to structural engineering materials. Resins are available in many colors, both transparent and opaque.

However, the most important role in the composition of resin is played by three components:

1. Monomers and prepolymers with appropriate reactivity,
2. Initiating system consisting of a photoinitiator or a complex system of photoinitiators and photosensitizers,
3. A UV blocker: a highly absorbing agent blocking unwanted excessive radiation of resin.

The mixture of monomers and prepolymers is mainly responsible for the viscosity of the liquid resin and gives the appropriate mechanical parameters to the final printout [53]. The reactivity of the monomer mixture and initiating system significantly affect the speed of the printing process and the need and time of the post-curing process [54]. The type, content, and parameters of the UV blocker and other polymerization inhibitors influence the maximum possible resolution. This effect is observed due to the fact that a certain critical concentration of initiating species (i.e., free radicals or protonic acid) in the illuminated monomer is required to effectively initiate the polymerization process. The higher the light intensity, the higher content of initiator, and the lower concentration of inhibitors and UV blockers, the quicker the initiation process occurs [55,56,57,58].

### 3.3. Photocuring 3D Printing Speed

A method to increase the speed of the 3D printing process, compared to the SLA, is the DLP method. Exposing the entire layer of photosensitive material instead of the SLA method, which illuminates individual paths in the material, allows for the possibility of significantly shortening the printing time, especially for projects containing large flat elements [59]. However, the resolution of the DLP method is considerably limited due to the technologies used in the construction of the printer. While the vertical accuracy of the printout is limited strictly by the aspects of the printing table mechanics, the horizontal resolution in the X–Y plane is limited by the size of a single micro-mirror in the DMD or pixel size in the method using an LCD screen. In the DLP method, which uses the projector’s layout for printing, the size of individual pixels increases as the displayed image is enlarged, which causes a decrease in resolution. Projecting an image smaller than the DMD or LCD matrix elements obtained by adjusting the optical system to display a reduced image size allows improving the resolution of printouts [60]. The printing speed depends on the light source used, its spectral characteristics and power, as well as the activity of the photoinitiator system and reactivity of printing resin. An additional limitation of the 3D printing speed is the printing process itself. As in the traditional SLA method, after exposure of a single layer lasting from a few to several seconds, the printer platform is lifted to detach the printout from the non-adhesive light-permeable film to introduce fresh liquid resin under the printout. Then the platform is lowered to a height with a given thickness of the printing layer, and the process is repeated [21].

When free radical polymerization is considered, the printing process can be accelerated by changing the standard light release film to an oxygen-permeable membrane. Oxygen penetrating through the membrane acts as a strong inhibitor of free radical photopolymerization on its surface, eliminating the need for detaching the print after exposure of every single layer. This is possible because the bottom of the vat resin has a thin layer of oxygen-saturated material that does not polymerize due to oxygen inhibition. Thus, it is possible to constantly raise the printout while being exposed. Because there is no need to constantly raise and lower the printer platform, this significantly reduces the printing time and also reduces distortion on printed components [61,62].

### 3.4. Resolving Power in the 3D Printing Processes

The use of a laser wavelength corresponding directly to the absorption of the photoinitiator limits the maximum print resolution according to the Abbe diffraction limit. The minimum resolvable distance d can be calculated from the Abbe diffraction limit as follows [44]:

(1) $$d=\frac{\lambda }{2\cdot NA}$$

where *NA* is the numerical aperture of a laser optical system, and *λ* is wavelength of the laser.

In the case of photopolymerization, where a high intensity of light is required to start the process, this condition can be overcome because polymerization starts effectively only in the center of the laser beam, where light intensity is highest [63].

**Figure 14:**
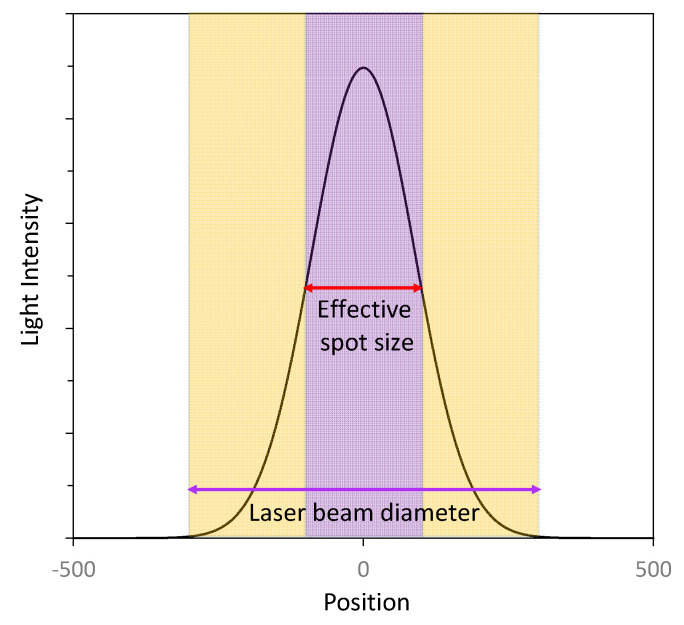
Distribution of light intensity across the laser beam.

Due to the fact that the collimated laser beam does not have uniform energy distribution along its radius, it is preferable to use inhibitors and UV blockers to obtain a resolution higher than the actual diameter of the laser beam, which can be observed in Figure 14. This increase in the resolution, however, has some limits, since with the rising concentration of UV blockers, the amount of light reaching deep in the sample is limited. With an increasing concentration of UV blockers, the effective diameter of the laser spot decreases rapidly, to a certain point, when a limit of the resolution is reached, and further addition of blocker results only in a significant reduction in the polymerization speed. In the case where a high content of UV blockers or polymerization inhibitors are present in the composition, the resulting reduction in effective lighting intensity necessitates increasing the exposure time of the sample and thus significantly extending the 3D printing process [64]. A side effect of the addition of UV blockers or polymerization inhibitors is also the limited maximum layer thickness that can be cured by incidental light [65].

Resolution limits resulting from the image projection method are difficult to exceed. Despite the continuous development of technology and the emergence of increasingly resolving DLP methods, the use of light in the UV/near VIS range in this technology limits the formation of sub-micrometric elements; however, DLP printing technology is currently one of the most popular 3D printing methods [65].

#### 3.4.1. Two-Photon Polymerization (TPP) as a High-Resolution 3D Printing Process

In order to bypass the restrictions in resolving power resulting from the use of light with a wavelength of near UV and the lower range of visible light, it was proposed to use the two-photon absorption effect [66]. In the process of one-photon absorption, the absorbance of material is linearly dependent on the intensity of lighting, while in the case of two-photon absorption, the dependence of absorption on the square of the intensity is observed. Due to this effect, in the focus of the laser beam, only a small central area is characterized by an intensity capable of initiating two-photon polymerization. The area with decent light intensity is therefore much smaller than when using one-photon absorption, which allows a significant improvement in resolution [67].

**Figure 15:**
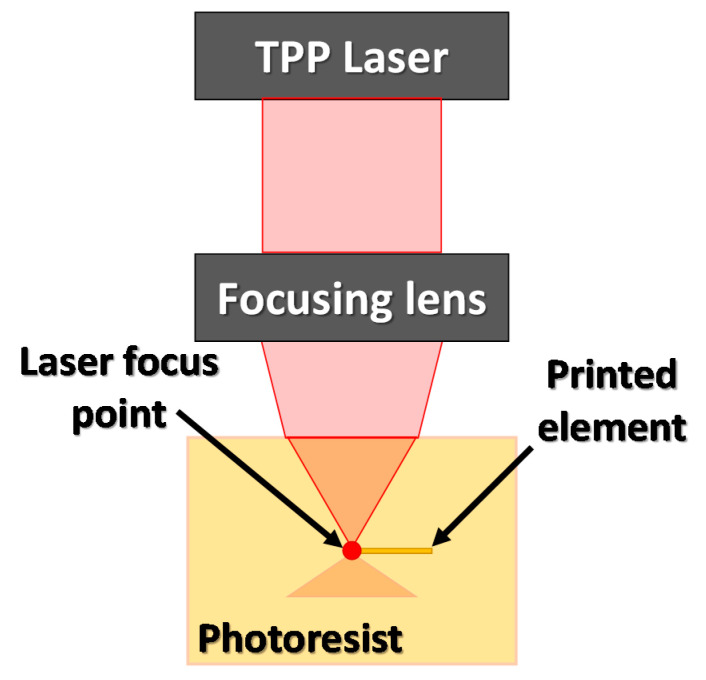
Two-photon polymerization (TPP) for direct writing in the photoresist scheme.

Due to the fact that the two-photon process is mediated by passing through a virtual electron state, which means that there is no energy state in the molecule corresponding to the absorption of a single photon, absorption of two photons must occur simultaneously. This means that the efficiency of the two-photon absorption process is very low. The phenomena of two-photon absorption can occur only when the sum of the energy of absorbed photons coincides with the energy difference between energetic levels of the absorbing medium. Therefore, the two-photon absorption process requires a very high intensity of lighting, and certain wavelengths with energy equal to half the energy of a single photon absorption band are favored [66]. The basic TPP scheme is shown in Figure 15.

Using traditional broad-spectrum light sources, providing such high energy concentrated in a small area resulted in thermal destruction of the sample, which is why pulse lasers were used for TPP processes. The use of lasers with a nano or even femtosecond pulse provides the sample with an effective dose of radiation, capable of initiating the TP polymerization process with a small amount of total energy transferred to the sample. This allows the photopolymerization process to take place without overheating of the sample [67]. The introduction of two-photon polymerization into the 3D printing process makes it possible to obtain 3D objects with a size equivalent of almost 1/100 of the wavelength of the light used. However, small changes in the regularity of photosensitive material and fluctuations in the intensity of the laser beam make it difficult to obtain such small objects. The development of special two-photon polymerization initiators allows an 80 nm resolution using an 808 nm infrared laser to be obtained. A reduction in the laser wavelength to 532 nm with a combination of the special initiating system contributes to a further increase in resolution to about 60 nm [44].

#### 3.4.2. Increasing Spatial Resolution of Two-Photon Polymerization (TPP) by the Application of a Photochromic Layer

**Figure 16:**
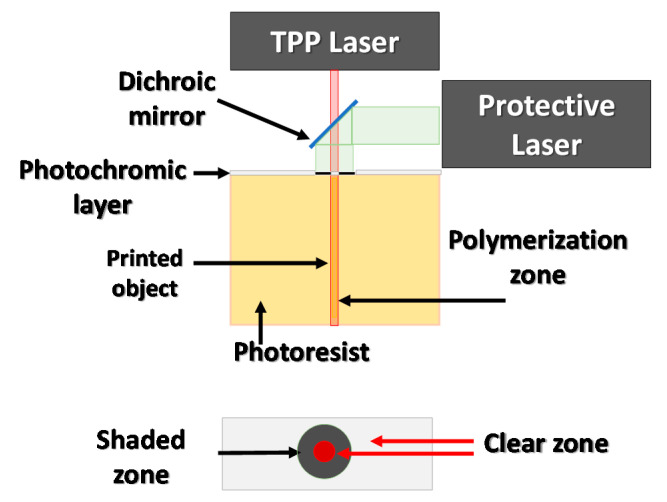
Restricting the curing area of a TPP laser by the application of a photochromic layer on top of the photoresist.

The resolution of the TPP method can be increased by restricting the width of the laser beam that reaches the photoresist. One of the proposed methods is to use a photochromatic layer that covers the surface of the photosensitive medium; such a system is shown in Figure 16. The photochromatic layer is illuminated with a laser to an appropriate wavelength, which causes a change in its permeability for a TPP laser with a different wavelength. The laser beam working on the photochromatic layer is formed around the area that is expected to undergo photopolymerization in such a way as to limit the effect of TRR laser radiation on the photosensitive material. This approach allows the minimal size of the printed elements to be significantly decreased, obtaining 36 nm printouts using a 325 nm wavelength laser [68].

#### 3.4.3. Increasing Spatial Resolution of the TPP by the Stimulated Emission Induced Depletion (STED) Method

**Figure 17:**
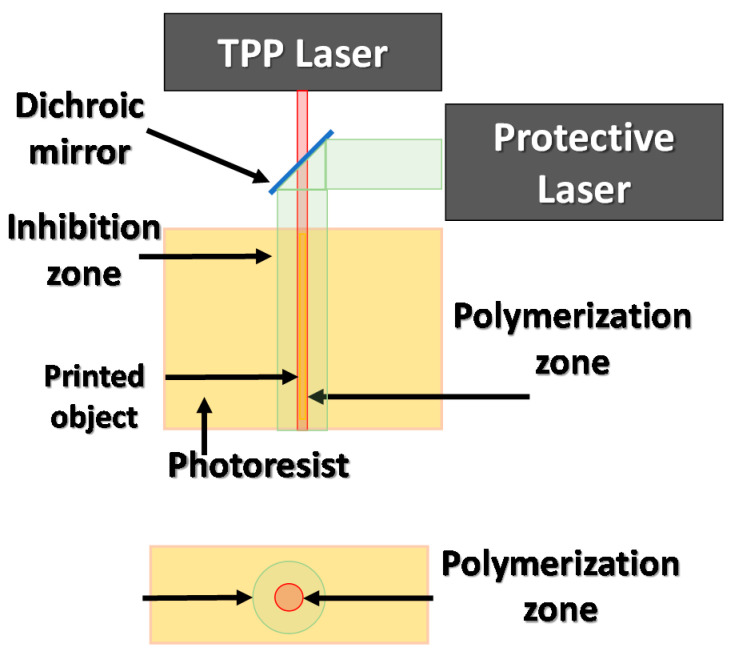
Stimulated emission induced depletion method scheme.

A further increase of the resolution in the two-photon photopolymerization is possible by modifying the photoresist exposure method to limit the effective beamwidth of the laser that reaches the sample to the minimum. For this purpose, the STED (stimulated emission induced depletion) method was developed based on the use of lasers with different wavelengths. In the STED method, the first laser beam enables the photopolymerization initiation; at the same time, the beam of the second laser beam encircles the first one, causing its inhibition. The polymer beam inhibiting polymerization is formed by a special optical system that is shaped like a ring. An initiating beam passes through the central part of the inhibitory beam ring, causing polymerization only in a specific area. The STED method enables the production of elements with a size of 9 nm when exposed to a laser wavelength of 800 nm [69,70]. The scheme of the STED method is presented in Figure 17.

#### 3.4.4. A Novel Solution for High Speed and Accuracy of TPP Printing

**Figure 18:**
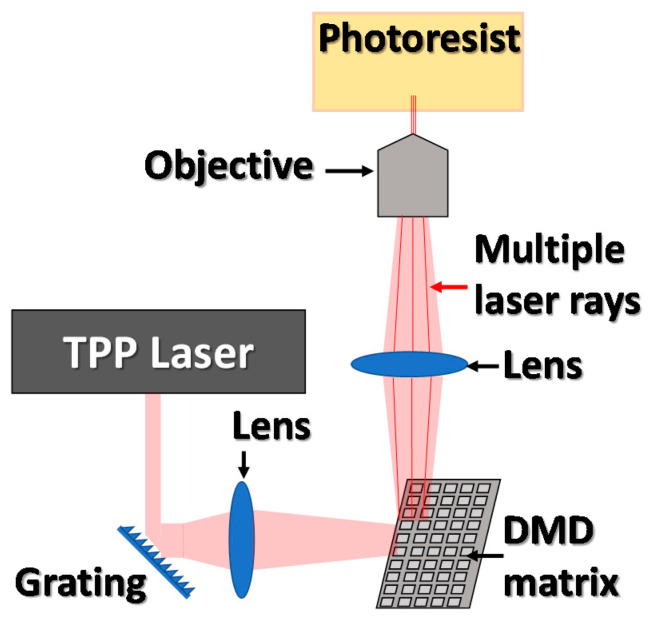
Visualization of the TPP coupled with a DMD matrix for simultaneous printing of multiple models.

Due to the very small curing area in two-photon polymerization, fabrication of even the smallest object can take days. The way to overcome this disadvantage is by changing the mechanism of the scanning device. Application of a digital micromirror device (DMD) on the laser optical path allows for the parallel fabrication of multiple objects with the same geometry. To achieve this, a single laser beam can be split into a number of rays by using a micro-lens array or by the application of proper diffraction elements. The arrangement of the TPP–DMD system is presented in Figure 18. With this approach, all manufactured elements are characterized by the same shape and geometry, which, depending on the application, can be a major inconvenience of the method [71].

Currently, the most promising area of research on two-photon polymerization is the digitalization of optics material used for driving the laser beam. The most forward-looking technique is the application of a computer-controlled liquid crystal matrix, which by precise control on liquid crystals (LC) inside of the LC device can properly drive a laser beam to form diffraction patterns corresponding to a printed element. The main limitation of this approach is that the power of a single laser beam is sheared between all focal points, so the power of a laser must be significant to efficiently initiate photopolymerization [72].

An indirect solution enabling a compromise between printing speed and accuracy in forming microstructures is by combining traditional 3D printing methods using UV/VIS light with printing by two-photon polymerization. In this technology, the first step is to form elements of larger sizes by irradiation of an appropriate mask. Then the microstructural elements are formed by using two-photon polymerization techniques. This approach allows the rapid production of structures with complex geometry containing sub-micrometric elements. Current research in this matter was conducted when exposing thin layers of photoresists, about 100 μm thick with a UV lamp through the mask, then conducting the two-photon polymerization process. By using this technique, elements of a 200 nm resolution were obtained. Formally, printouts created in this method can be treated as 2D objects in terms of traditional lithography, while the structure formed by two-photon polymerization bears the features of a 3D structure. The authors describe this process as the 2.5D process [73,74].

### 3.5. Functional Materials for High-Precision and Innovative Additive Manufacturing Solutions

Photosensitive materials used in 3D printing, in addition to their basic properties, can be modified to obtain unique parameters. The basic way to modify the resins is to change their chemical composition, which includes both the content of monomers and photoinitiating systems [75]. Modifications to the basic resin’s composition affect mainly the mechanical and optical properties of the compositions; however, a much wider range of performance parameters can be reached by the preparation of composite materials [76,77].

#### 3.5.1. Mechanical and Electrical Properties of 3D Printed Composites

The introduction of solid materials into a liquid monomer mixture requires their proper distribution in the resin matrix. The mixed materials must be compatible with each other to prevent aggregation and structural irregularities inside the printed composites. For this purpose, solid materials are subjected to surface modifications, the purpose of which is to attach functional groups at their surface, which interact strongly with the monomers or are capable of forming chemical bonds during polymerization of the resin [78]. In other words, the solid material introduced into the photosetting resin is incorporated into the polymer structure [79]. The literature describes examples of improving the mechanical parameters of resins to which the addition of multi-wall carbon nanotubes (MWCNTs) were introduced. To enable an accurate dispersion of the modifier, as well as to improve the mechanical properties of the material, carbon nanotubes were first subjected to acid washing, followed by chemical modification introducing thiol moieties capable of reacting with the acrylate resin on the MWCNT surface. This method was successfully applied in conjunction with two-photon polymerization, which led to the obtaining of structures of sub-micrometric sizes [80,81]. Composite materials that use modified carbon nanotubes as fillers show—in addition to improving the mechanical properties of the composite—increased electrical conductivity of the crosslinked material. However, to achieve significant electrical conductivity, it is necessary to thoroughly percolation nanotubes through the resin, which allows the creation of a three-dimensional network of connections between individual nanoparticles and the polymer matrix. This resin-conductive filler system has been successfully used for printing micro and nanostructural electronic components such as resistors or capacitors [82].

#### 3.5.2. Magnetic 3D Printing Nanocomposites

Materials with magnetic properties are used for the contactless operation of moving parts in devices not only at the macro scale but also at the micro scale. The magnetic elements interacting with the magnetic field form the basis for the operation of devices such as loudspeakers, galvano mechanisms, and DC motors. They can be used to manipulate small particles, constitute movable elements of solenoid valves, and enable the precise control of drug delivery systems. Photocurable materials used in 3D printing, as well as two-photon polymerization, do not have magnetic properties. To enable the interaction of polymeric materials with an applied magnetic field, dispersed micro or nanomagnetic particles can be introduced into such a system [83]. The most popular type of particles used to produce magnetic composites are magnetite (Fe_3_O_4_) nanoparticles, but the fineness of the particle powder is not enough to obtain sub-micrometric composite structures. Aggregation of particles in the photosensitive material is also a negative phenomenon; in this case, it reduces the resolution, and in the printing of nanometric structures leads to a significant deterioration in the mechanical properties of the composite. For that reason, Fe_3_O_4_ particles were modified by the introduction of acrylate moieties onto their surfaces. Such an operation enabled the formation of chemical bonds on the filler-resin line during polymerization. The introduction of such modified particles in the amount of 2% enabled a homogeneous composite with magnetic properties to be obtained in the TPP process. A similar approach made it possible to obtain composite nanostructures based on SU-08 resin with 4% magnetic nanoparticles (MNP) [84]. Composites with magnetic nanoparticles have also been obtained in the form of hydrogel materials. MNP hydrogel at 1% composite based on polypropylene glycol diacrylate was successfully printed with a spatial resolution of 110 nm. Another group obtained similar materials based on methacrylated gelatin. Magnetic nano hydrogel structures have been proposed as intelligent drug carriers driven by the magnetic field [81,85].

#### 3.5.3. 3D Printing Advanced Optical Materials

The process of 3D printing using photosetting materials has also been applied for the production of optical elements. The simplest optical systems obtained in the 3D printing process are lenses [86]. By using transparent resins with an appropriate refractive indexes, it is possible to obtain lenses of any shape, size, and operational parameters. On the macro-scale, the lenses can be successfully printed using the SLA method, while on a sub-micrometer scale, two-photon polymerization must be applied. In addition to simple lenses, the 3D printing process allows the simultaneous preparation of more complex optical systems. In the two-photon photopolymerization process, an optical system consisting of a mono-material lens mounted on the tip of an optical fiber equipped with an internal hollow structure was obtained. After the printing process, the hollow structure was filled with photocurable material with a different refractive index to form a reflective structure placed at a 45° angle to the lens [87].

Elements with much smaller spatial dimensions, printed by the TPP technique, are photonic crystals. Such systems are made up of alternately arranged fragments of materials with significantly different refractive indexes. The photo-curable materials usually have a refractive index not exceeding n = 1.7. To obtain photonic crystals with the desired parameters, nanoparticles of optically active materials are introduced into liquid photosetting resins. As optically active materials, nanopowders of chalcogenogen glasses, lead selenide, lithium niobate, or metallic nanoparticles synthesized in situ can be used [88].

An interesting approach was presented by Duan et al., which produced photon crystals based on urethane acrylates with the use of titanium (IV) acrylate complexes. Titanium oxide (TiO_2_) nanoparticles were generated in situ by simultaneous hydrolysis and heat treatment of the material. The ready material showed a decrease in transmittance by 82% compared to the initial one, which proved the formation of TiO_2_ nanoparticles. Similar materials were obtained by direct addition of TiO_2_ nanoparticles to a liquid matrix, which turned out to be a better solution for industrial applications than synthesis in situ. Both methods gave comparable n values for ready material—around 1.65 with a TiO_2_ content of 2% [89,90].

The introduction of a significant amount of ceramic fillers or metal powders into photosetting resins can also be used for 3D printing of elements, which after annealing will provide the properties of porous ceramic or metal structures [91]. For this purpose, zirconium oxide in the form of micro and nanoparticles was used, which, due to chemical surface modifications, was a reactive filler of the photosetting resin. The high content of ceramic filler combined with the photochemical curing mechanism enabled 3D printing in SLA technology. The printed elements were burned out at a high temperature and ceramic zirconium materials were obtained, which, according to the authors, may open the way to an application in dentistry [51].

## 4. Conclusions

Additive manufacturing methods are widely used in all industries requiring rapid prototyping. Thanks to the implementation of 3D scanning and computer-aided design methods, translation of the computer project into a real 3D printed model is now easier than ever before [92]. Prototype or 3D printed product development is now possible on a scale from several nanometers up to multimeter 3D prints. Depending on chosen method, fabricated objects will differ in shape accuracy and general resolution starting from methods with low resolution methods like FDM with up to 100 μm resolution, to SLA and DLP method with accuracy up to of several micrometers, and to nanoscale 3D printing with TPP techniques. On the other hand with rising accuracy, printing speed slows down to level of days needed for fabrication of tiny element with nanoscopic resolution. Thanks to recent development in two photon polymerization, printing speed can be radically shortened by fabrication of multiple objects simultaneously, but for now, this approach demand more expensive and more powerful light sources, and advanced optics.

The methods of 3D printing allow models to be made from materials as diverse as flexible rubber-like materials to specific materials with conductive, magnetic or specialized optical properties, and the range of available materials rises annually. During the development of additive methods, materials with possible biological applications and hydrogel materials were also obtained. The major advantages and disadvantages of each described method are presented in Table 1. The choice of method used to make the appropriate model brings the consequences of an indirect selection of material resources, printing accuracy, maximum resolution, and the need for printing support structures, as well as printing time and the physical properties of the finished model.

## Figures and Tables

**Table 1:** Comparison of described methods.

Family	Method (Light Source)	Advantages	Disadvantages
Stereolithography	Direct SLA (laser 365 to 405 nm) [26]	Oldest and well-developed method with multiple materials available. Fair resolution.	Low printing rate, resolution limited by the size of the laser beam and further with Abby diffraction limit
	SLA–TPP (laser 532–1064 nm) [66,67]	Great resolution. The rising availability of materials.	Slow process—even the smallest models created in days.
	SLA–TPP with a dichromic layer (laser 532–1064 nm) [68]	Outstanding resolution of prints (a few nm).	Slow process—even the smallest models created in days. The necessity for the application of an additional photochromic layer on top of the photoresist.
	SLA–STED (laser 532–1064 nm) [69,70]	Outstanding resolution of prints (few nm).	Slow process—even the smallest models created in days. Tailoring of initiating–inhibiting systems needed for all materials.
	SLA–TPP–DMD (laser 532–1064 nm) [71]	Great resolution. Possible fabrication of multiple objects at the same time.	Underdeveloped method.
	SLA–TPP–Liquid crystal devices (laser 532–1064 nm) [72]	Outstanding resolution. Possible fabrication of multiple objects at the same time. Faster than SLA–TPP–DMD	Underdeveloped method.
Fused deposition modeling	FDM (-) [93]	Great availability of filaments, moderate mechanical properties of printouts.	Low-resolution method. Can cause deformation of printouts during the process.
Digital Light Processing	DLP–DMD projector (HID lamp/LED lamp) [94]	High resolution for small models. High precision for small models.	Bigger models decrease resolution.
	LCD–DLP (405–450 nm LED lamp) [21]	High resolution. Resolution independent on model size	Low light intensity reaching resin vat, only highly reactive resins can be used.
Inkjet	Binder Jetting (HID lamp/LED lamp) [38,42,95]	Numerous materials available, relatively quick printing process.	Low resolution. Poor mechanical properties of green printouts.
	Inkjet(HID lamp/LED lamp) [38]	High resolution, multiple materials available. Smooth surface finish.	The high cost of printer and materials. Only low viscous materials can be printed.
	Multijet (HID lamp/LED lamp) [44,45,46]	High resolution, multiple materials available. Possibility of printing with multiple materials at the same time. Possible creation of colorful printouts. Smooth surface finish.	The high cost of printer and materials. Only low viscous materials can be printed.

Fair resolution—hundreds of micrometers to tens of micrometers. High resolution—tens of micrometers to micrometers. Great resolution—single micrometers to hundreds of nanometers. Outstanding resolution—tens of nanometers to several nanometers.
